# Occurrence of hemodynamic changes following administration of rocuronium in a dog presenting for an ophthalmic procedure

**DOI:** 10.1002/vms3.1531

**Published:** 2024-07-02

**Authors:** Melissa Calus, Kirk A. Muñoz

**Affiliations:** ^1^ Veterinary Medical Center, College of Veterinary Medicine Michigan State University East Lansing Michigan USA; ^2^ Department of Veterinary Clinical Sciences, College of Veterinary Medicine The Ohio State University Columbus Ohio USA

**Keywords:** diphenhydramine, histamine, hypotension, rocuronium, tachycardia

## Abstract

A 10‐year‐old, neutered male, Golden Retriever dog presented for surgical correction of a descemetocele. Acepromazine (0.02 mg/kg) and methadone (0.5 mg/kg) were administered intramuscularly for sedation, propofol (2 mg/kg) and midazolam (0.2 mg/kg) were administered intravenously for anaesthetic induction and isoflurane in oxygen was utilised for anaesthetic maintenance. Rocuronium (0.5 mg/kg), a neuromuscular blocking agent, was administered intravenously to facilitate central positioning of the eye for surgery. Within 10 min of rocuronium administration, the dog became tachycardic and hypotensive. Hemodynamic aberrations did not resolve with initial interventions but were successfully mitigated with the administration of diphenhydramine (0.8 mg/kg) intravenously. The dog remained stable throughout the remainder of the procedure and experienced a smooth and uneventful recovery. While it is difficult to confirm that the hemodynamic changes observed in this clinical case resulted solely from administration of rocuronium, the observance of the cardiovascular changes, timing of events and response to therapy suggest that rocuronium elicited a histamine response that was successfully treated with diphenhydramine.

## Graphical abstract

This case report outlines the cardiovascular changes that occurred surrounding the administration of rocuronium, a non‐depolarising neuromuscular blocking agent, to a healthy dog during an ophthalmic procedure. Within 10 min of administration of rocuronium, the dog became tachycardic and hypotensive. Hemodynamic aberrations did not resolve with initial interventions but were successfully mitigated with the administration of diphenhydramine at 0.8 mg/kg intravenously, indicating that rocuronium likely elicited a histamine response.

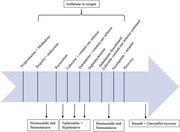

## 1 INTRODUCTION

Neuromuscular blocking agents (NMBA) are commonly incorporated into anaesthetic plans to facilitate muscle relaxation in humans and dogs (Dugdale et al., 2002). Non‐depolarising NMBAs agents are divided into two categories: benzylisoquinolinium compounds, such as atracurium and cisatracurium or amino steroids such as rocuronium. Drugs of either category contain a quaternary ammonium ion, which have been shown to cause anaphylactic reactions and histamine release (Naguib et al., 1995). The quaternary ammonium is recognised by immunoglobulin E (IgE) in the body as a foreign molecule, which leads to significant histamine release and may result in vasodilation and subsequent hypotension and compensatory tachycardia (Mills et al., 2014). Studies in humans have shown that benzylisoquinolinium compounds have been associated with a histamine response as a result of their prominent quaternary ammonium ion, and can result in hypotension and tachycardia (Lieberman, 1998). Benzylisoquinolinium compounds are further reported to cause a 200% and higher increase in histamine plasma concentration (Naguib et al., 1995). Rocuronium has been reported to not cause significant changes in cardiovascular variables or plasma histamine concentrations (Naguib et al., 1995). While an animal is under anaesthesia, the major negative clinical outcome of a histamine response is vasodilation with subsequent hypotension, and in some case bronchoconstriction. A hypotensive state, in humans and dogs, is expected to result in a compensatory tachycardia, an attempt to mitigate the decrease in blood pressure (Chrusch et al., 1999; Moss & Rosow, 1983; Shmuel & Cortes, 2013). Other clinical signs sometimes seen with histamine release are urticaria and pyrexia (Shepherd, 2003).

There is a dearth of information in veterinary medicine literature regarding an amino steroid structure, such as rocuronium, causing a histamine response and methods to manage such an event (Savarese et al., 2000). This case describes an apparent histamine response after intravenous (IV) administration of rocuronium in a dog while under anaesthesia, which was successfully managed with the administration of diphenhydramine.

## 2 CASE HISTORY

A 10‐year‐old, neutered male, Golden Retriever dog weighing 38.6 kg presented to the ophthalmology service at XXX to undergo surgical correction of a descemetocele. On initial physical examination, the dog's heart rate (HR) was 120 beats per min (bpm) with an elevated respiratory rate. Remaining vitals, complete blood count and biochemistry panel were unremarkable.

Acepromazine at 0.02 mg/kg and methadone at 0.5 mg/kg were administered intramuscularly (IM), achieving moderate sedation to facilitate intravenous catheter placement. Approximately 30 min later, anaesthesia was induced with propofol at 2 mg/kg, and midazolam at 0.2 mg/kg as a co‐induction agent. The dog was then intubated with an 11‐mm internal diameter endotracheal tube. Anaesthesia was maintained via isoflurane in oxygen at 2.5% for 15 min, later decreased to 2% due to stability of the dog. Lactated Ringer's solution (LRS) was provided IV at a rate of 5 mL/kg/h. Maropitant at 1 mg/kg and ondansetron at 0.2 mg/kg were slowly administered IV immediately after induction, for their antiemetic and antinausea properties. An antibiotic, cefazolin (22 mg/kg), was administered slowly IV just after induction and every 90 min thereafter intraoperatively. Indirect blood pressure monitoring was performed every 5 min using a Mindray Passport multiparameter monitor (Mindray). The blood pressure cuff was placed on a hindlimb and was measured to be 40% of the circumference of the limb.

**FIGURE 1:**
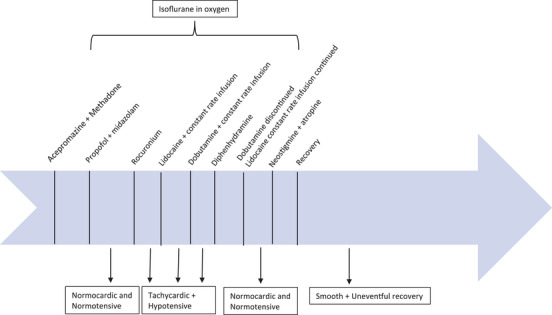
A 10‐year‐old male neutered Golden Retriever dog presented for surgical correction of a descemetocele in the dog's right eye. The dog became tachycardic and hypotensive within 10 min of administration of rocuronium, which did not respond to lidocaine and dobutamine interventions. Shortly after diphenhydramine was administered intravenously the dog became normocardic and normotensive, and remained stable for the remainder of the procedure. Recovery from anaesthesia was smooth and uneventful.

Immediately after induction, the dog was stable with a systolic arterial blood pressure (SAP) of 100 mm Hg, diastolic arterial blood pressure (DAP) of 45 mm Hg and mean arterial blood pressure (MAP) of 60 mm Hg. Other vitals such as temperature (100.2°F), HR (100 bpm), respiratory rate (12 breaths per min [brpm]) and end‐tidal CO_2_ (45 mm Hg) were within normal limits. Shortly thereafter, the patient displayed intermittent periods of apnoea and a mechanical ventilator (Hallowell EMC) was added to maintain a respiratory rate of 8 brpm and a tidal volume of 400 mL. A peripheral nerve stimulator (SunMed LLC) was used to monitor the train of four (TOF) response due to the use of an NMBA. The nerve stimulator was attached near the peroneal nerve; the positive lead was placed lateral to the tibial tuberosity and the negative lead placed 3‐inches distal to the positive lead on the lateral aspect of the stifle. Twenty minutes post‐induction, rocuronium at 0.5 mg/kg was administered slowly IV to achieve paralysis of the ocular muscles facilitating central positioning of the eye for surgery. Within 10 min of rocuronium administration, a zero out of four TOF count was observed and the neuromuscular blockade was considered to be successful. However, the patient became tachycardic, with a HR of 160 bpm, and hypotensive with a SAP of 50 mm Hg, DAP 15 mm Hg and a MAP of 35 mm Hg. A bolus of lidocaine at 2 mg/kg was administered slowly IV to mediate any potential painful stimulus causing the tachycardia, with the intent that it would reduce the HR and allow more time for cardiac filling. The dog's HR remained elevated at 145 bpm and MAP continued to decrease to 20 mm Hg. An LRS fluid bolus (10 mL/kg, 380 mL total volume) was administered, a dobutamine constant rate infusion (CRI) was started at 7 mcg/kg/min, as well as a lidocaine CRI of 50 mcg/kg/min, all administered IV. Dobutamine was included for its positive inotropic effect, while lidocaine was utilised for its antinociceptive effect (Ortega & Cruz, 2011; Tuttle & Mills, 1975). Ten to fifteen minutes after these initial interventions, vitals were as follows: HR was 150 bpm, end‐tidal CO_2_ was 42 mm Hg, MAP was 45 mm Hg and SpO_2_ was 99%. With no marked improvement in HR and MAP, diphenhydramine at 0.8 mg/kg was administered IV, in the unlikely instance that the rocuronium had elicited a histamine response. Within 15 min of diphenhydramine administration, the dog's vitals improved to a HR of 110 bpm, end‐tidal CO_2_ of 52 mm Hg, MAP of 65 mm Hg and SpO_2_ of 99%. The patient was then weaned off of the dobutamine CRI but the lidocaine CRI was continued to provide additional analgesia (Ortega & Cruz, 2011; Smith et al., 2004). The remainder of the procedure was uneventful with vital parameters remaining within normal ranges. At the end of surgery, neostigmine at 0.04 mg/kg and atropine at 0.02 mg/kg were administered IV to antagonise the effect of the NMBA. Once spontaneous breathing resumed, mechanical ventilation was discontinued and the inhalant was turned off. Thirty minutes later, the dog recovered smoothly and uneventfully. Post‐operative vitals were as follows: temperature of 100.4°F, HR of 94 bpm, panting at a rate of 64 brpm, thoracic auscultation detected no abnormalities and mucous membranes were noted to be pink with a capillary refill time of less than 2 s (Figure 1). At this time, all monitoring equipment was removed as the animal was awake and moving around. The recommendation was made to not administer rocuronium to this dog in any future events.

## 3 DISCUSSION

This is the first report of diphenhydramine successfully treating an apparent histamine response, manifested as tachycardia and hypotension, suspected to be elicited by administration of rocuronium in a dog. A study by Küls et al. (2016) reported the suspicion of a non‐allergic reaction to rocuronium, with the affected dog displaying tachycardia, hypertension and bronchoconstriction as compared to the tachycardia and hypotension observed in this case. While acepromazine, which has α_1_‐adrenoceptor antagonistic properties, and methadone were used as premedicants in this case, drugs which could have contributed to the hypotension and resultant tachycardia, hemodynamic changes did not occur until shortly after the administration of rocuronium (Hall et al., 2001). Within 10 min of rocuronium administration, the blockade was successful; however, the dog was now markedly tachycardic and hypotensive. Due to placement of surgical drapes and warming devices, cutaneous changes, such as erythema and urticaria, that may have occurred alongside a suspected histamine response were not observed. While epinephrine is the recommended treatment for anaphylaxis, the signs seen in this case were not very severe, the dog did not show any signs of bronchoconstriction, and was unlikely to be in anaphylactic shock (Kroigaard et al., 2007). Diphenhydramine, an H_1_‐receptor antagonist, is often used pre‐emptively to suppress hypersensitivity responses to drugs, food, blood products and other allergens (Helgeson et al., 2021). Since rocuronium is not reported to cause a histamine response in dogs, these cases are not typically pre‐treated with an antihistamine, and as such when the HR and blood pressure initially changed they were not thought to be associated with the NMBA.

In this case, the dog's HR was 1.6 times higher and the blood pressure was 1.7 times lower at 10 min following NMBA administration versus post‐induction vitals. Initial suspicion was that the tachycardia was the result of noxious surgical stimulation. However, initial drug interventions were unsuccessful in resolving the tachycardia and hypotension. Since the tachycardia was paired with hypotension rather than hypertension, and no improvement was seen with the initial interventions, the changes were then believed to have been the result of a histamine response rather than response to a surgical stimulus. The correlation of tachycardia and hypotension with administration of rocuronium, along with the resolution of the adverse clinical signs after diphenhydramine was administered, is highly suggestive of a histamine response.

## 4 CONCLUSION

Diphenhydramine appeared to successfully treat the adverse changes seen shortly after administration of rocuronium in this dog. While there were confounding factors in determining the cause of the marked tachycardia and hypotension, it is highly likely that these changes occurred due to a histamine response associated with the use of rocuronium (Mertes & Volcheck, 2015; Testa et al., 2021).

## AUTHOR CONTRIBUTIONS

**Melissa Calus**: Conceptualisation; methodology; writing—original draft and writing—review and editing. **Kirk A. Muñoz**: Conceptualisation; investigation; methodology; resources; supervision; writing — original draft and writing — review and editing.

## CONFLICT OF INTEREST STATEMENT

The authors declare no conflicts of interest.

### ETHICS STATEMENT

None.

### PEER REVIEW

The peer review history for this paper is available at https://publons.com/publon/10.1002/vms3.1531.

## Data Availability

Data available on request from the authors.
